# Regulation of cellular proteostasis via mitochondrial protein import

**DOI:** 10.1098/rstb.2025.0271

**Published:** 2026-10-01

**Authors:** Ngoc G. T. Nguyen, Jian Li

**Affiliations:** New York Medical College, Valhalla, NY 10595, USA

**Keywords:** mitochondria, protein import, proteostasis, stress response

## Abstract

Mitochondria are the powerhouse of the cell, playing vital roles in energy production and metabolism. Most mitochondrial proteins are encoded in the nuclear DNA and must be synthesized in the cytosol before being transported into the appropriate mitochondrial compartments. Mitochondrial protein import is not only essential for mitochondrial biogenesis but also a crucial regulatory step in mitochondrial proteostasis surveillance and stress response. Additionally, defects in mitochondrial protein import lead to mislocalization of precursor proteins to the cytosol, disrupting cytosolic proteostasis and contributing to various human diseases. This review summarizes recent findings demonstrating that mitochondrial protein import is a key regulator of cellular proteostasis.

This article is part of the Theo Murphy meeting issue ‘ProteostaSys: a systems view of proteostasis’.

## 1. Introduction

Mitochondria play essential roles in energy metabolism, biosynthesis, calcium homeostasis and programmed cell death in eukaryotic cells [1–4]. While mitochondria retain a compact genome that encodes a limited number of proteins, about 99% of the more than 1000 mitochondrial proteins, including the intra-mitochondrial translation machinery, are encoded in the nuclear genome, synthesized by cytosolic ribosomes and imported into the mitochondria [5,6]. Mitochondrial protein import not only is essential but also has critical regulatory roles in mitochondrial biogenesis. Many mitochondrial precursor proteins are metastable and aggregation-prone, requiring molecular chaperones, such as cytosolic Hsp70, to maintain them in a soluble, largely unfolded state for transport. Defects in mitochondrial protein import result in the retention and accumulation of precursor proteins in the cytosol, thereby disrupting cellular proteostasis [7,8] and contributing to human diseases [9,10]. This review summarizes recent discoveries from yeast, invertebrate models and mammals, highlighting the mitochondrial protein import machinery as a regulatory hub for mitochondrial biogenesis and protein quality control, and its impacts on both mitochondrial and cytosolic proteostasis.

Mitochondria are double-membraned organelles that use diverse pathways recognizing sequence or structural signals to sort precursor proteins into four compartments: the outer mitochondrial membrane (OMM), the inner mitochondrial membrane (IMM), the intermembrane space (IMS) and the matrix. Most imported proteins pass through the translocase of the outer membrane (TOM). The exceptions are the α-helical outer membrane proteins inserted into the OMM by the mitochondrial import (MIM) complex in yeast [11]. The other imported proteins first traverse the OMM into the IMS via the TOM complex before being sorted to distinct locations. The sorting and assembly machinery complex inserts β-barrel proteins from the IMS into the OMM [12], and the TIM22 complex, one of the translocases of the inner membrane, inserts hydrophobic proteins, such as metabolite carriers, into the IMM [13]. In addition, the mitochondrial IMS import and assembly (MIA) pathway couples import with oxidative folding, thereby promoting the retention of precursor proteins containing multiple cysteines in the IMS by forming internal disulfide bonds [14]. Finally, the presequence translocase of the inner membrane, the TIM23 complex, is responsible for importing most mitochondrial proteins that contain mitochondrial targeting signals (MTSs) enriched with positively charged residues into the matrix or the IMM (figure 1). About 60% of imported mitochondrial proteins contain an N-terminal cleavable MTS [15], and the TOM–TIM23 pathway may recognize additional proteins via an internal MTS [16]. TIM23 opens laterally and releases the precursor proteins containing a hydrophobic transmembrane segment into the IMM [17,18]. For precursor proteins destined for the matrix, translocation requires cooperation between the TIM23 complex and the presequence translocase-associated motor (PAM) complex. Molecular details of the diverse mitochondrial protein import pathways have been discussed in previous reviews [2–4,19,20]. Here, we focus on the TOM–TIM23 pathway, which transports most metabolic enzymes to the IMM and the matrix and works closely with protein quality control systems in both the mitochondria and the cytosol to maintain cellular proteostasis.

**Figure 1.**
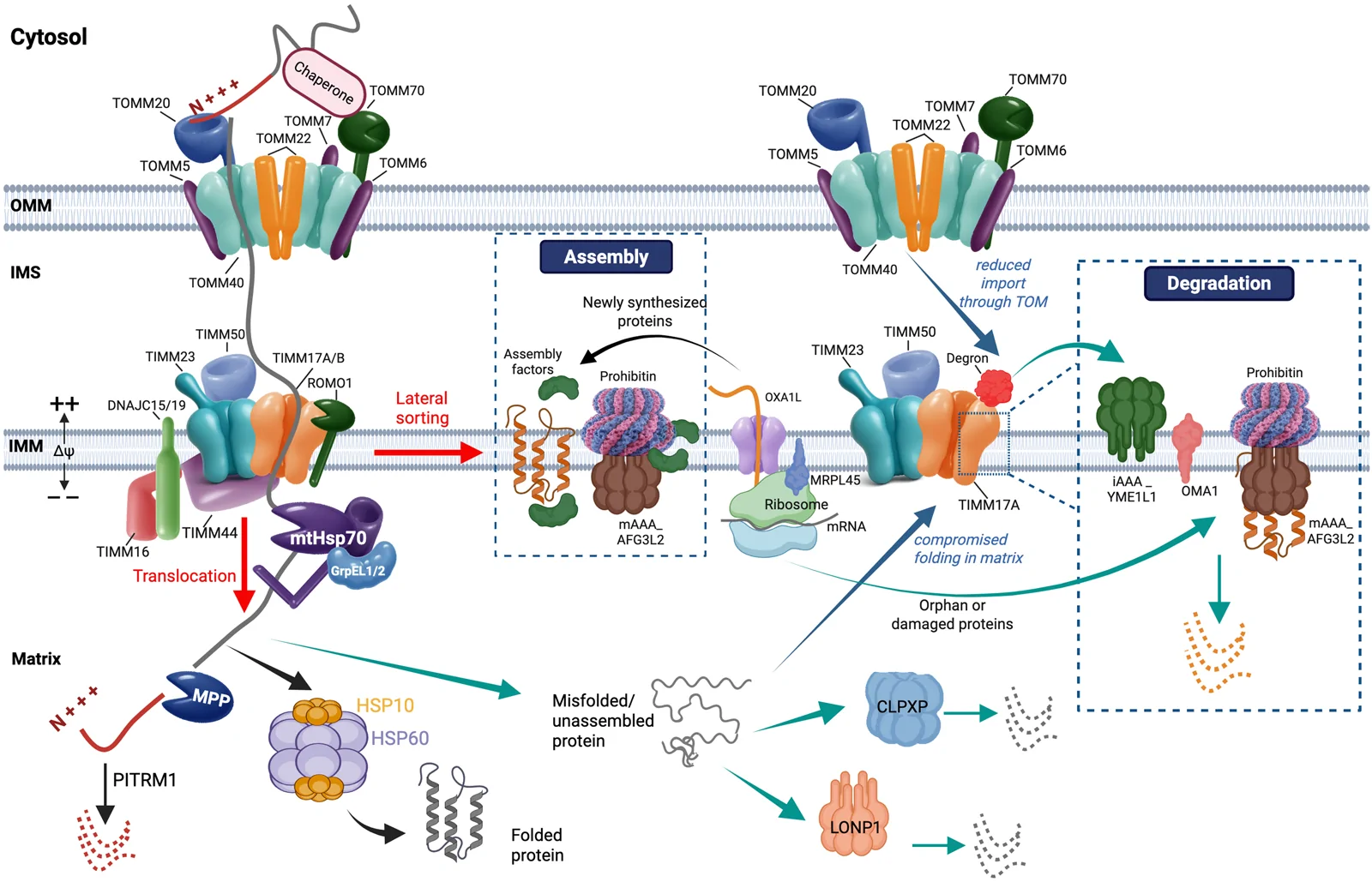
TOM–TIM23 import pathway in mitochondrial biogenesis, protein quality control and stress sensing in human cells. (Left) During mitochondrial biogenesis, precursor proteins containing a mitochondrial targeting signal (MTS) are imported by the TOM–TIM23 pathway. TOMM70 recruits cytosolic chaperones to keep precursor proteins soluble and transport-ready. Matrix-destined proteins are pulled through the translocation path formed by TIMM17A or TIMM17B in the TIM23 translocase by the PAM complex. The PAM complex binds to the TIM23 translocase via TIMM44 and powers translocation through ATP hydrolysis by mtHsp70, assisted by its cochaperones DNAJC15/19, TIMM16 and GrpEL1/2. Upon import into the matrix, the N-terminal MTS is cleaved from the precursor protein by mitochondrial processing peptidase (MPP) and further degraded by peptidases such as PITRM1. MPP-processed proteins are folded by the mitochondrial chaperonin complex formed by HSP60 and its cochaperone HSP10. The TIM23 translocase can also open laterally via ROMO1, releasing precursor proteins containing a hydrophobic transmembrane segment into the inner mitochondrial membrane (IMM), including many subunits of oxidative phosphorylation (OXPHOS) complexes. These nuclear-DNA-encoded OXPHOS subunits are assembled with mitochondrial ribosome-synthesized subunits, which are co-translationally inserted into the IMM by OXA1L, at the prohibitin–mAAA complex when assembly factors are available. Right: Imported proteins are monitored by the mitochondrial protein quality control system. Misfolded proteins in the matrix are degraded by the AAA proteases, LONP1 and CLPXP. The membrane-bound matrix-facing AAA proteases (mAAA), AFG3L2 homo-hexamer or AFG3L2 and SPG7 hetero-hexamer, the intermembrane space (IMS)-facing AAA protease (iAAA), YME1L1, together with OMA1 peptidase, remove unassembled and unwanted proteins from the IMM. The short-lived TIMM17A is an emerging stress sensor for translation attenuation in the cytosol, clogged TOM and compromised protein folding in the matrix. Under those stress conditions, TIMM17A is degraded by YME1L1, thereby reducing the protein import capacity of the TIM23 translocase. Red arrows: protein sorting; black arrows: mitochondrial biogenesis; cyan arrows: protein degradation; blue arrows: stress sensing by TIMM17A. OMM, outer mitochondrial membrane; Δψ, inner membrane potential.

## 2. Quality control during mitochondrial protein import

Cryo-electron microscopy structures suggest that the TOM complex contains twin protein-conducting channels formed by a dimer of the β-barrel Tom40, tethered by two Tom22 subunits at the centre and stabilized by three small Tom proteins (Tom5, Tom6 and Tom7) on the periphery [21–23] (figure 1; table 1). In addition to its structural role, Tom22 works with Tom20 as the primary receptor for the MTS [88]. Tom70 was considered the receptor for presequence-less proteins. Recent work suggests that Tom70’s primary function is to serve as a docking site for cytosolic chaperones, thereby increasing the solubility of precursor proteins at the mitochondrial surface [29]. Consistent with this, Tom70 also facilitates the transport of presequence-containing proteins recognized by Tom20 but containing aggregation-prone mature domains [30]. After passing through TOM, the MTS is recognized by the Tim50 receptor of the TIM23 translocase [37] (figure 1; table 1). The essential Tim23 and Tim17 subunits form the core of the TIM23 translocase. The negatively charged residues of Tim17 near the IMS side of the bilayer attract the positively charged residues in the MTS and are critical for initiating translocation [18]. Recent structural and biochemical analyses in yeast suggest that Tim17, rather than the Tim23 subunit, forms the protein translocation path, and that the non-essential subunit Mgr2 (ROMO1 in humans) shields the lateral cavity of Tim17, serving as the gatekeeper for protein sorting into the IMM [17,18]. The PAM complex, which associates with the TIM23 translocase, uses the power of ATP hydrolysis by mitochondrial Hsp70 (mtHsp70) and its cochaperones to pull presequence proteins into the matrix. Within the PAM complex, the adaptor protein Tim44 recruits mtHsp70 to TIM23 and precisely positions the DnaJ protein Pam18 (DNAJC15 and DNAJC19 in humans) and the J-like protein Pam16 (TIMM16 in humans) for mtHsp70 activation [41], while Mge1 (GrpEL1 and GrpEL2 in humans) acts as the nucleotide exchange factor (NEF) to regulate the mtHsp70 ATPase cycle [48,89].

A recent structural study reveals that a TOM–TIM23 supercomplex forms and spans the mitochondrial inner and outer membranes during transport [90]. This supercomplex provides a platform that orchestrates protein translocation and quality control in both the cytosol and mitochondria. TOMM70 recruits cytosolic chaperones to the mitochondrial outer membrane, rendering precursor proteins soluble and transport-competent, thereby minimizing cytosolic proteotoxicity associated with precursor protein retention and aggregation. The PAM complex uses the mtHsp70 chaperone to couple protein import to folding in the mitochondrial matrix: mtHsp70 captures presequence proteins as they emerge from the TIM23 translocase and hydrolyses ATP to power translocation and the initial stages of folding. After MTS cleavage, partially folded proteins are handed over to the mitochondrial chaperonin complex, consisting of HSP60 and HSP10, for further folding and maturation [49]. Before reaching the stable native conformation and being assembled into the designated protein complex, newly imported mitochondrial proteins are hypersensitive to stress and susceptible to misfolding and aggregation [91]. The long filamentous phenotype (LON) proteases and caseinolytic proteases (CLPs) degrade misfolded and orphan proteins, playing an essential role in protein quality control in the matrix [3].

A unique challenge in mitochondrial biogenesis and proteostasis is the proper assembly of the oxidative phosphorylation (OXPHOS) complexes, which contain subunits encoded by both mitochondrial DNA (mtDNA) and the nuclear genome. The mtDNA-encoded OXPHOS proteins typically contain multiple transmembrane domains and are co-translationally inserted into the IMM by the oxidase assembly protein OXA1 (OXA1L in humans) [92]. The fate of mitochondria-translated polypeptides depends on the availability of their nucleus-encoded partners and on assembly efficiency. Import or assembly defects trigger degradation of unassembled OXPHOS proteins by the matrix-facing mAAA or IMS-facing iAAA proteases (figure 1; table 1). An essential player in IMM protein quality control is the prohibitin–mAAA complex, which resides near the polypeptide tunnel exits (PTEs) of mitoribosomes and interacts with both the import machinery and OXPHOS assembly factors [93,94]. The prohibitin complex is proposed to serve as an early assembly hub that bridges mitochondrial translation, protein import and OXPHOS complex assembly at the IMM. Prohibitin forms a large ring-like structure in the membrane that reduces mAAA protease activity [95–98], providing the opportunity for newly synthesized, mitochondria-encoded subunits to interact with their assembly factors and be incorporated into the OXPHOS complexes with imported, nucleus-encoded subunits. However, a delay in assembly would prolong the interaction between newly synthesized polypeptides and the prohibitin–mAAA complex, thereby increasing their degradation by the mAAA protease. This mechanism provides early molecular triage for mitochondrial-translated proteins, directing them either to be assembled into the OXPHOS complex or to be degraded, thereby preventing the accumulation of unassembled, orphan proteins at the IMM.

## 3. Regulation of mitochondrial biogenesis through the import machinery

Mitochondrial biogenesis responds dynamically to nutritional, developmental and environmental cues. Mitochondrial protein import depends on the membrane potential, generated by the electron transport chain during oxidative phosphorylation, and consumes ATP. In the meantime, the mitochondrial protein import machinery senses cellular demands for respiration and responds to stress signals to adjust mitochondrial biogenesis and energy production. Mitochondrial protein import can affect mitochondrial biogenesis by regulating transcription. For example, during *Caenorhabditis elegans* larval development, animals activate the stress-responsive ATFS-1 transcription factor to upregulate nucleus-encoded mitochondrial genes involved in mitochondrial translation, protein import, quality control, OXPHOS complex assembly and others, thereby expanding the mitochondrial network [99]. ATFS-1 contains a weak MTS as well as a nuclear localization sequence (NLS), and its transcriptional activity is regulated by its subcellular localization [100]. Once imported into mitochondria, ATFS-1 is degraded by the Lon protease (LONP-1 in *C. elegans*). During development, when cytosolic synthesis of mitochondrial proteins exceeds import capacity, ATFS-1’s weak MTS is outcompeted, leading to a fraction of ATFS-1 being excluded from mitochondria and localized to the nucleus, thereby activating its transcriptional programme which promotes mitochondrial protein import and biogenesis.

Mitochondrial protein import also regulates mitochondrial translation. Transcript-specific mechanisms have been identified that control mitochondrial translation in response to the levels of imported OXPHOS proteins. In yeast, feedback control is mediated by translational activators that promote translation initiation of the corresponding transcripts only when newly synthesized mtDNA-encoded subunits are assembled into OXPHOS complexes [101–104]. For example, cytochrome *b* (COB) requires assembly factors Cbp3–Cbp6 at the mitoribosomal PTE to stabilize the newly synthesized protein and assemble it into Complex III. When Cbp3–Cbp6 are engaged in cytochrome *b* assembly, COB mRNA and its translational activators, Cbs1 and Cbs2, are bound to the IMM protein Mrx4 and are not available for translation initiation. Once Cbp3–Cbp6 are free from assembly, they bind to Mrx4, releasing Cbs1, Cbs2 and the bound COB mRNA for a new round of translation [105,106]. This feedback loop allows mitochondrial translation to respond to cytosolic protein synthesis and import during metabolic shifts and stress [107], providing sufficient mtDNA-encoded proteins to match the demands for OXPHOS complexes while preventing the accumulation of unassembled IMM proteins. Humans use a conceptually consistent but mechanistically distinct strategy to coordinate mitochondrial translation, protein import and OXPHOS complex assembly [108]. During the biogenesis of cytochrome *c* oxidase (Complex IV), assembly factors C12ORF62 and MITRAC12 associate with the mtDNA-encoded COX1 co-translationally. COX1 translation elongation stalls in the absence of COX4, the first nucleus-encoded subunit that associates with newly synthesized COX1. Co-translational interaction with assembly factors primes the COX1 ribosome–nascent chain complex for OXPHOS complex assembly and adjusts COX1 translation in response to the import of its nucleus-encoded partners.

Not only the efficiency of mitochondrial protein import but also the abundance and composition of the import machinery respond to physiological and stress signals. The human TIM23 translocase contains one of the two paralogous Tim17 proteins: TIMM17A or TIMM17B. Although TIMM17A and TIMM17B have very similar structures [109], their biogenesis and stability are differentially regulated [110]. TIMM17A is short-lived and stress-responsive, while TIMM17B is more stable and is proposed to perform housekeeping functions. Compared with TIMM17B, TIMM17A contains a unique C-terminal degron recognized by the iAAA protease YME1L1 [32] (figure 1). YME1L1-mediated TIMM17A degradation is induced upon cytosolic translation attenuation, as in the integrated stress response (ISR), in which cap-dependent translation initiation is suppressed owing to eIF2α phosphorylation [33]. A recent study shows that clogging TOM translocase is sufficient to expose the TIMM17A degron in the unoccupied TIM23 complexes for degradation [32]. Therefore, YME1L1-regulated TIMM17A stability serves as a mechanism that adjusts mitochondrial protein import capacity in response to the rate of cytosolic protein synthesis and the demands for protein transport. Proteolysis by YME1L1 also plays an essential role in cellular adaptation to hypoxia and nutrient starvation by limiting mitochondrial biogenesis [34]. YME1L1 proteolytic activities are regulated by a lipid signalling cascade mediated by mTORC1 and its downstream phosphatidic acid phosphatase LIPIN1, which alters the phosphatidylethanolamine (PE) levels in mitochondrial membranes. The mechanism by which decreased PE during hypoxia and nutrient starvation promotes YME1L1-dependent proteolysis is yet to be explored. The YME1L1–TIMM17A regulatory axis also senses proteostasis in the mitochondrial matrix, as suggested by a recent study [35]. TIMM17A levels decrease when protein folding in the matrix is compromised upon knockdown of the mitochondrial chaperonin complex, which, in turn, reduces mitochondrial protein import and translation to rebalance the demands for protein folding with the levels of chaperones. Therefore, TIMM17A regulates mitochondrial protein import in response to proteostasis in both the cytosol and mitochondria.

Interestingly, distinct half-lives and stress sensitivities are also observed in other paralogous proteins within human mitochondrial protein import machinery, including the PAM18 proteins, DNAJC15 and DNAJC19, and the NEFs for mtHsp70, GrpEL1 and GrpEL2 [5]. Biochemical experiments have identified that TIM23 complexes that harbour TIMM17A preferentially associate with the short-lived DNAJC15, while TIMM17B-containing translocase preferentially associates with the stable DNAJC19 [31]. The interaction between DNAJC15 and TIMM17A is mediated by the unique N-terminal IMS-exposed region of DNAJC15 and the C-terminus of TIMM17A, where the degron of TIMM17A is located. A recent study has shown that mitochondrial deficiencies, such as OXPHOS defects, activate the OMA1 peptidase to cleave DNAJC15 at its N-terminus, rendering it susceptible to degradation by mAAA proteases [111] (figure 1). This provides an additional mechanism to reduce mitochondrial protein import and biogenesis under stress. Like TIMM17B, GrpEL1 is much more stable than its paralogue and plays an essential role in protein translocation to the matrix [112] by serving as the primary NEF for mtHsp70 [113]. Conversely, GrpEL2 has a short half-life and is redox-regulated [112]. Whether GrpEL2 directly regulates protein transport, as its yeast orthologue, Mge1, does in oxidative stress, remains unclear. Finally, despite its short half-life, TIMM17A is overexpressed in cancers, promoting cell proliferation and associated with poor prognosis [114], highlighting its potential role as a driver of mitochondrial biogenesis in rapid cell growth. Similarly, PAM16/TIMM16, which regulates the recruitment of DNAJC15 and DNAJC19 to the TIM23 translocase, is also overexpressed in cancers [115].

## 4. Surveillance mechanisms coping with mitochondrial protein import stress

Mitochondrial protein import is energy-demanding and stress-sensitive, yet many precursor proteins are aggregation-prone. Import defects cause the cytosolic accumulation of mitochondrial precursor proteins, posing a particular challenge to the cellular proteostasis network. Overexpression of variants of ANT1, an ADP/ATP carrier protein, clogs the TOM complex and leads to mitochondrial precursor overaccumulation stress (mPOS), featuring impaired ribosomal biogenesis and induction of aggresome-like structures in the cytosol [7,10]. mPOS induces cell death and underlies muscle atrophy and neurodegeneration [9,10]. A genetic suppressor screen revealed that reducing protein synthesis or enhancing cytosolic protein degradation attenuates mPOS, indicating that cellular toxicity primarily results from disruption of cytosolic proteostasis [7]. Similarly, accumulation of mitochondrial precursors in the cytosol is observed when the MIA pathway is impaired. To cope with proteotoxicity, cells inhibit protein synthesis and activate the ubiquitin–proteasome system (UPS) to degrade cytosol-retained precursor proteins, a process termed the unfolded protein response activated by the mistargeting of proteins (UPRam) [8].

In addition to clearance of mistargeted precursors, surveillance mechanisms are in place to recover clogged protein translocases when mitochondrial protein import stalls (figure 2; table 1). One mechanism involves the cytosolic AAA-ATPase Cdc48 in yeast and VCP (valosin-containing protein, also known as p97) in humans, which are recruited to the TOM complex by the adaptor protein Ubx2 in yeast and UBXD8 (also known as FAF2) in humans to power ATP hydrolysis-coupled unfolding and extraction of translocation-arrested precursors for proteasomal degradation [70,71]. This pathway, termed mitochondrial protein translocation-associated degradation (mitoTAD), uses the same AAA-ATPase and adaptor protein as the endoplasmic reticulum (ER)-associated degradation (ERAD) pathway, which eliminates misfolded or unassembled proteins from the ER [116]. Mitochondria use dynamic ubiquitylation and deubiquitylation to monitor precursor protein import [67,117]. In yeast, the E3 ubiquitin ligase Rsp5 associates with the TOM complex and ubiquitylates translocation-arrested precursor proteins, while the deubiquitylase Ubp16 removes ubiquitin that may be erroneously added to a precursor by Rsp5 [67]. March5 and USP30 are the mammalian E3 ubiquitin ligase and deubiquitylase pair that regulate precursor import at the TOM complex [117]. TOM20 is a substrate of USP30, and the abundance of the TOM complex decreases in USP30 knockout mice, suggesting an additional role for USP30-dependent deubiquitylation in maintaining TOM complex stability. The adaptor proteins Ubx2 and UBXD8 are localized to the OMM by a hydrophobic membrane anchor (MA) and recruit the ATPases Cdc48 and VCP, respectively, via their UBX (ubiquitin regulatory X) domains [70,71]. The adaptor proteins also contain a UBA (ubiquitin-associated) domain that may facilitate binding to ubiquitylated client proteins [118,119]. A recent study identified a ubiquitin-binding helix (UBH) in UBXD8 adjacent to the UBX domain that accelerates VCP's ATP hydrolysis and enhances its unfolding and extraction efficiency [71]. Finally, the yeast Ufd1–Npl4 (mammalian UFD1L–NPLOC4) heterodimer serves as a cofactor for Cdc48 by capturing polyubiquitin chains and unfolding an initiator ubiquitin, which triggers the unfolding and extraction of the remaining ubiquitin chains and the attached protein by Cdc48 [72].

**Figure 2.**
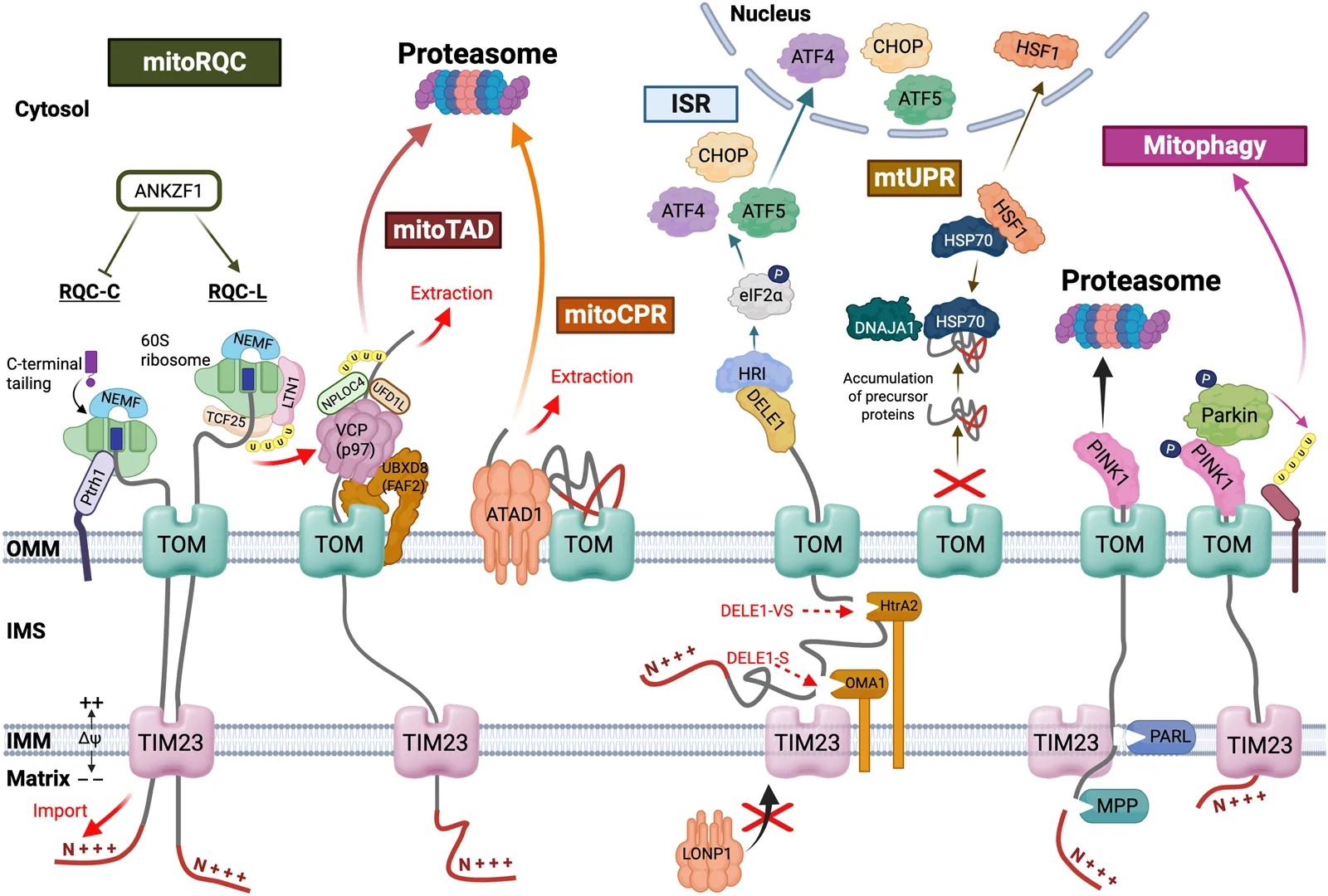
Surveillance mechanisms and stress signalling pathways that respond to mitochondrial protein import deficiency in human cells. Mitochondria-associated ribosomal quality control (mitoRQC) removes polypeptides that are co-translationally targeted to the mitochondria from a stalled ribosome. Ribosome stalling first triggers the splitting of the 80S ribosome (not shown). NEMF recognizes the remaining 60S–peptidyl-tRNA complex and recruits the E3 ubiquitin ligase LTN1 to ubiquitylate the nascent polypeptides (RQC-L pathway, L for LTN1). The endonuclease ANKZF1 releases the nascent polypeptides from tRNA, which are extracted by the VCP/p97 AAA-ATPase for proteasomal degradation. While ANKZF1 promotes RQC-L, it inhibits NEMF-mediated C-terminal tailing (RQC-C pathway), which increases the aggregation propensity of nascent polypeptides and challenges mitochondrial proteostasis upon release into mitochondria by the peptidyl-tRNA hydrolases Ptrh1. The inhibitory role of ANKZF1 in RQC-C is inferred from its yeast orthologue, Vms1. Mitochondrial protein translocation-associated degradation (mitoTAD) recovers clogged translocases from import-arrested precursor proteins. The adaptor protein UBXD8/FAF2 at the outer mitochondrial membrane (OMM) recruits the AAA-ATPase VCP/p97 to ubiquitylated, translocation-arrested precursor proteins. Assisted by the cofactors UFD1L and NPLOC4, VCP/p97 uses the power of ATP hydrolysis to unfold and extract the stalled precursor protein for proteasomal degradation. The mitochondrial compromised protein import response (mitoCPR) was identified in yeast as a mechanism that inducibly recruits the AAA-ATPase Msp1 to TOM to extract proteins from clogged translocases for degradation. Although the stress response pathway is not fully conserved, ATAD1, the mammalian homologue of Msp1, has been reported to remove translocation-arrested precursors from TOM when mitochondrial protein import is impaired. The integrated stress response (ISR) is induced upon mitochondrial protein import deficiency. Instead of being imported into the matrix of healthy mitochondria and degraded by LONP1, DELE1 is cleaved by OMA1 or HtrA2 in response to protein import defects. DELE1 fragments released into the cytosol interact with and activate the HRI kinase, which phosphorylates eIF2α, leading to a global reduction in protein synthesis and increased expression of the stress-responsive transcription factors ATF4, ATF5 and CHOP. The mitochondrial unfolded protein response (mtUPR) upregulates mitochondrial chaperones via HSF1 in response to compromised protein folding in the matrix. Mitochondrial stress causes non-imported precursor proteins to accumulate in the cytosol and increases reactive oxygen species. Oxidized DNAJA1 enhances the recruitment of HSP70 to precursor proteins, thereby relieving HSP70’s inhibition of HSF1. Mitophagy is triggered by the import arrest of PINK kinase. Under non-stress conditions, PINK is imported and inserted into the inner mitochondrial membrane (IMM), where it is cleaved by the PARL protease and released into the cytosol for proteasomal degradation. Import arrest stabilizes PINK at the OMM, where it phosphorylates and activates the E3 ubiquitin ligase Parkin to initiate mitophagy.

While mitoTAD operates constitutively as a surveillance mechanism, additional quality control factors are required when clogging-prone proteins exceed mitoTAD's capacity. In budding yeast, the lateral sorting pathway of the TIM23 translocase is a rate-limiting step in mitochondrial protein import. Overexpression of proteins containing a bipartite signal, an MTS and a hydrophobic segment that directs them to the IMM could saturate the TIM23 complex and elicit a transcriptional stress response called the mitochondrial compromised protein import response (mitoCPR) [73]. mitoCPR is mediated by the transcription factor Pdr3. A recent study revealed a surprising mechanism for Pdr3 activation by Mge1, the cochaperone and NEF for mtHsp70 [120]. Under mitochondrial protein import stress, Mge1 is excluded from the mitochondria and translocated into the nucleus, where it binds to and activates Pdr3 via its MTS. *CIS1* is among the most highly induced genes in mitoCPR. The Cis1 protein recruits the AAA-ATPase Msp1 to the TOM complex by binding to Tom70, where they mediate the clearance of unimported precursor proteins from the mitochondrial surface [73]. Although Cis1 is not conserved in mammals, ATAD1, the mammalian homologue of Msp1, has been shown to remove translocation-arrested precursors from TOM when mitochondrial protein import is impaired [74], likely being recruited through an alternative mechanism.

Although most mitochondrial proteins are synthesized in the cytosol and imported post-translationally, proximity-specific ribosomal profiling in yeast estimates that about one-third of mitochondrial proteins are co-translationally targeted [121]. Translation defects and faulty mRNA templates can lead to ribosome stalling. Translationally arrested ribosomes are recognized by the ribosome-associated protein quality control (RQC) pathway, which routes aberrant nascent polypeptides to the proteasome for degradation and recycles ribosomes [122]. The RQC system also monitors co-translationally targeted mitochondrial proteins and is tailored to remove stalled polypeptides from translocases while minimizing the import of toxic proteins into mitochondria via a process termed mitoRQC (figure 2; table 1) [65]. Ribosome stalling first triggers the splitting of the 80S ribosome, which retains the peptidyl-tRNA with the 60S subunit [122]. The RQC component Rqc2 in yeast (NEMF in mammals) recognizes the 60S–peptidyl-tRNA complex and recruits the E3 ubiquitin ligase Ltn1 to ubiquitylate the nascent polypeptide. Rqc1 (TCF25 in mammals) guides Ltn1 to synthesize ubiquitin Lys48-linked chains, which subsequently recruit the Cdc48 (VCP in mammals) complex to extract nascent peptides that are already engaged in mitochondrial translocation for proteasomal degradation in the cytosol [61,65]. Nascent-peptide release from the P-site tRNA is mediated by tRNA cleavage by the Vms1 endonuclease (ANKZF1 in mammals), which leaves the 3′-terminal CCA nucleotides of the tRNA attached to the nascent chain [61]. If the exposed nascent peptides lack lysine residues for Ltn1-mediated ubiquitylation, yeast Rqc2 adds a carboxy-terminal alanine and threonine (CAT) tail to the stalled nascent chain in the absence of an mRNA template, exposing lysine residues that are otherwise sequestered in the ribosome exit tunnel [62]. Similarly, mammalian NEMF can extend stalled polypeptides, with alanine being the most frequently incorporated amino acid in the C-terminal tail [63]. The C-terminal extension increases the accessibility of lysine residues in stalled polypeptides to the canonical Ltn1-dependent RQC (RQC-L, L for Ltn1). However, if ubiquitylation fails owing to inactivation of Ltn1 or because the stalled polypeptide lacks lysine residues, the addition of the CAT tail or a poly-Ala tail increases the aggregation propensity of the nascent proteins [63,123]. Those polypeptides with C-terminal tails can be released by the peptidyl-tRNA hydrolases Pth2 in yeast and Ptrh1 in mammals, which hydrolyse the peptidyl-tRNA ester linkage [68,124]. In mammals, if the polypeptides are released to the cytosol, the poly-Ala tail can be recognized by the Pirh2 or CRL2(KLHDC10) E3 ligases as a C-terminal degron, which directs the polypeptides to proteasomal degradation (RQC-C, C for C-end rule) [61,63]. However, if the stalled polypeptides are already engaged in MIM by TOM–TIM23, the C-terminal tail-containing nascent chains released by Pth2/PTRH1 are likely pulled into the matrix by mtHsp70. Those aggregation-prone polypeptides impose a heavy burden on the mitochondrial protein quality control system, sequestering chaperones and requiring protease activity for degradation [65,125]. To address this challenge to mitochondrial proteostasis, Vms1 counteracts Rqc2, thereby reducing CAT tailing in peptides that are eventually released into the mitochondrial matrix [65]. In addition, a recent report suggests that slowing mitochondrial protein import generally protects mitochondria from proteotoxicity associated with CAT tailing, even in the absence of Ltn1, potentially by increasing the likelihood that stalled nascent polypeptides are extracted by cytosolic chaperones [68].

## 5. Stress signalling elicited by mitochondrial protein import deficiency

Mitochondrial protein import efficiency is sensitive to OXPHOS activities that generate the inner membrane potential and ATP, and it requires coordination of the import machinery and protein quality control pathways. Impairment of OXPHOS and perturbations in mitochondrial genome, translation, protein folding and degradation that disrupt mitochondrial proteostasis converge on decreased mitochondrial protein import, thereby inducing the mitochondrial unfolded protein response (mtUPR) (table 1) [126–131]. The mtUPR was discovered in mammalian tissue culture cells [132], and its response to mitochondrial protein import deficiency was established in studies of the MTS- and NLS-containing transcription factor ATFS-1 in *C. elegans*. As in mitochondrial network expansion during development, ATFS-1 activation under mitochondrial stress is triggered by exclusion of ATFS-1 from mitochondria owing to impaired protein import [86]. Nuclear-localized ATFS-1 reprogrammes transcription of mitochondrial and non-mitochondrial genes to help cells recover from mitochondrial dysfunction [133]. In the mtUPR, ATFS-1 induces expression of mitochondrial chaperones and proteases to restore mitochondrial proteostasis. The mtUPR also shifts ATP production from mitochondrial OXPHOS to cytosolic glycolysis by upregulating glycolytic genes and downregulating components of OXPHOS complexes. ChIP-seq and transcriptomic analyses suggest that ATFS-1 regulates expression of OXPHOS genes in both nuclear and mitochondrial DNA, either directly by binding to promoters or indirectly [133]. However, the mechanism by which ATFS-1 represses OXPHOS genes remains to be elucidated. Finally, ATFS-1 upregulates expression of the mitochondrial protein import machinery, including components of the TOM and TIM23 translocases. This helps preserve a certain level of import capacity before the membrane potential is restored [134], thereby allowing chaperones, proteases and other mitochondrial proteins induced by the mtUPR to enter mitochondria. It is noteworthy that other transcription factors and chromatin remodellers are also involved in the mtUPR in *C. elegans* and act in concert with ATFS-1, as summarized in previous reviews [135,136].

Reduced mitochondrial protein import also plays critical roles in eliciting protective stress responses to mitochondrial dysfunction in mammals. It has been reported that ATF5, a mammalian orthologue of ATFS-1 with both nuclear and mitochondrial localization, responds to mitochondrial stress in a manner similar to ATFS-1 and contributes to the mtUPR [137]. ATF5 is expressed at low levels in most cells under non-stress conditions and has a short half-life [138,139]. Additional transcription factors and mechanisms are clearly involved in the mammalian mtUPR. Compromised protein folding in the mitochondrial matrix activates the mtUPR through HSF1 [87], the central transcriptional regulator of the heat shock response that copes with unfolded proteins in the cytosol [140]. In the mtUPR, HSF1 is activated in response to import defects that cause accumulation of mitochondrial precursors in the cytosol [87] (figure 2). In addition, mitochondrial reactive oxygen species contribute to HSF1 activation by oxidizing DNAJA1, a cochaperone of cytosolic HSP70. DNAJA1 oxidation enhances the recruitment of HSP70 to mitochondrial precursors, releasing HSF1 from HSP70-mediated inhibition and enabling HSF1 nuclear localization. Mitochondrial protein import deficiency also relays mitochondrial stress to the cytosol, thereby activating the ISR, which remodels global translation in response to diverse stress conditions (figure 2; table 1). Functional genomics screens identified DELE1 as a key regulator of the ISR under mitochondrial stress [82,83]. DELE1 is imported into the matrix of healthy mitochondria and degraded by the LONP1 protease [141]. Mitochondrial stress impairs DELE1 import and activates the OMA1 peptidase to cleave DELE1, releasing a C-terminal fragment of DELE1 (DELE1-S, S for short) to the cytosol [82,83]. DELE1-S interacts and activates HRI, one of the kinases that phosphorylates the translation initiation factor eIF2α in the ISR. While eIF2α phosphorylation represses cap-dependent translation globally, it selectively activates the expression of stress-responsive transcription factors, including ATF4, ATF5 and CHOP [82,83,142]. OMA1 is dispensable for DELE1 processing in certain cell types, where the HTRA2 peptidase functions in place of OMA1 to cleave import-arrested DELE1 in the IMS and release DELE1-VS (VS for very short) into the cytosol, thereby inducing the ISR [84].

In addition to triggering stress responses that help restore mitochondrial proteostasis, mitochondrial protein import deficiency also regulates mitophagy, which removes unsalvageable mitochondria via autophagy. The mitophagy pathway senses mitochondrial defects through the import of PINK kinase (figure 2; table 1). PINK is imported into healthy mitochondria and inserted into the IMM, where it is cleaved by the rhomboid protease PARL, resulting in its release into the cytosol for rapid proteasomal degradation [77,143]. Loss of mitochondrial membrane potential causes PINK import arrest, which stabilizes PINK at the OMM associated with the TOM–TIM23 supercomplex [144]. Several TOM complex subunits and the TIM23 complex facilitate PINK binding at the TOM complex, among which Tom7 and the TIM23 complex are shown to prevent cleavage of PINK by the stress-activated OMA1 [144–147]. The accumulation of PINK at the OMM triggers its autophosphorylation and activation [148]. Consequently, PINK phosphorylates ubiquitin that is attached to OMM proteins, recruiting the E3 ubiquitin ligase Parkin and priming it for PINK-mediated phosphorylation [75,76,149,150]. Activated Parkin marks mitochondria for removal by mitophagy through ubiquitylation of OMM proteins. Like mitochondrial depolarization, mitochondrial protein misfolding also causes PINK import arrest. Misfolded proteins in the matrix dissociate the PAM complex from the TIM23 complex, thereby activating the mitophagy pathway without loss of membrane potential [151].

## 6. Concluding remarks

Mitochondrial protein import plays multifaceted roles in cellular proteostasis. It is an essential step in mitochondrial biogenesis, sorting >99% of the mitochondrial proteome into the correct sub-mitochondrial compartments and influencing mitochondrial translation through OXPHOS assembly and feedback regulation. It works closely with the mitochondrial protein quality control system, including chaperones, assembly factors and proteases, to assist protein folding and assembly and to triage misfolded and orphan proteins for degradation. Growing evidence suggests that the mitochondrial protein import machinery functions as a sensor for both OXPHOS demands and mitochondrial stress, adjusting import rates in response to cytosolic translation levels and mitochondrial proteostasis capacity. Conversely, mitochondrial protein import defects lead to the accumulation of precursor proteins in the cytosol, posing a significant challenge for proteostasis and contributing to neurodegenerative diseases and muscle atrophy. Multiple surveillance mechanisms have been identified to cope with import stress and restore clogged translocases. Given the high dependence on OXPHOS activity and mitochondrial proteostasis, mitochondrial protein import also serves as a sentinel of mitochondrial dysfunction, eliciting protective stress responses or mitophagy to repair or remove damaged mitochondria.

While research over the past decade has significantly broadened our understanding of mitochondrial protein import and the import stress response pathways in both cytosolic and mitochondrial proteostasis, it also raises many open questions for future studies. It is intriguing that the human mitochondrial protein import machinery contains paralogous proteins with dramatically different half-lives. Emerging evidence suggests that the short-lived TIMM17A and DNAJC15 proteins are stress-sensitive and that their levels or functions are elevated in cancer. Changes in either TIMM17A or DNAJC15 remodel the steady-state mitochondrial proteome and mitochondrial functions in cancer cells [35,111]. As TIMM17A is the preferred partner of DNAJC15 and forms the translocation path in the TIM23 translocase, determining whether overexpression of TIMM17A in cancer alters mitochondrial protein import capacity and/or import selectivity, and whether it alters cytosolic precursor retention, may provide insights into cancer treatment. Furthermore, mitochondria regulate protein import capacity through the mtUPR in response to increased cytosolic translation during development and to perturbations of mitochondrial proteostasis. It is unclear whether only the amplitude of the mtUPR, or the targets as well, differ under physiological and stress conditions. Although the same transcription factor may regulate the mtUPR in both cases, such as ATFS-1 in *C. elegans*, the transcriptional programmes may still differ owing to differences in the chromatin environment and/or cofactors, as observed for many stress-responsive transcription factors, including HSF1 [152]. Finally, although this review focuses on the roles of mitochondrial protein import in cellular proteostasis, its impact on animal physiology extends to the organismal level, involving cell-non-autonomous regulation. While mitochondrial stress is generally harmful to cells, mild perturbation of OXPHOS in *C. elegans* promotes longevity by activating the mtUPR, which preserves mitochondrial protein import with a compromised membrane potential [134], reorganizes chromatin structures [153] and improves cytosolic proteostasis by enhancing HSF1 activity [154]. Interestingly, studies in *C. elegans* and mammals have shown that neurons with mitochondrial dysfunction secrete signalling factors, termed mitokines, that activate the mtUPR in a cell-non-autonomous manner in peripheral tissues [155–158]. Future work is required to elucidate the molecular mechanisms by which mitokines influence mitochondrial protein import and the mtUPR activation in peripheral tissues and fully understand their roles in mitochondrial physiology during ageing.

**Table 1.** Regulators of mitochondrial protein import by the TOM–TIM23 pathway and cellular responses to mitochondrial protein import stress. IMM, inner mitochondrial membrane; OMM, outer mitochondrial membrane. *C. elegans*, *Caenorhabditis elegans*.

functional group	yeast	human	molecular functions
the translocase of the outer mitochondrial membrane (TOM)	Tom5	TOMM5	small Tom proteins regulate TOM complex assembly and stability; Tom5 and Tom7 may be involved in sorting precursor proteins through the Tom40 channel [22,24,25]
	Tom6	TOMM6	
	Tom7	TOMM7	
	Tom20	TOMM20	Tom20 recognizes the presequences and directs precursors to Tom40 [26]
	Tom22	TOMM22	Tom22 is involved in presequence binding and Tom40 dimer assembly [27,28]
	Tom70	TOMM70	Tom70 serves as a docking site for cytosolic chaperones and facilitates the import of precursor proteins containing aggregation-prone mature domains [29,30]
	Tom40	TOMM40	Tom40 dimer forms the protein translocation channels of the TOM complex [21,22]
the translocase of the inner mitochondrial membrane (TIM)	Tim17	TIMM17A	Tim17 forms the translocation path in the TIM23 complex [17,18]; TIMM17B is essential for the housekeeping function [31]; TIMM17A is short-lived and responsive to proteotoxic stress [32–35]
		TIMM17B	
	Tim23	TIMM23	Tim23 plays a structural role, providing a platform for the binding of Tim17, Tim44 and Tim50 [17,36]
	Tim50	TIMM50	Tim50 is the putative presequence receptor of the TIM23 complex [36–38]
	Mgr2	ROMO1	Mgr2 and ROMO1 are not essential for general import; Mgr2 shields the lateral cavity of Tim17 and regulates protein sorting into the IMM [17,39,40]
	Tim44	TIMM44	Tim44 serves as a scaffold for association and precise positioning of mtHsp70 and its cochaperones to the Tim23 : Tim17 translocase [41]
	Pam16/Tim16	TIMM16	Pam16 forms a heterodimer with Pam18, important for Pam18’s association with the Tim23 : Tim17 translocase and modulating Pam18 activity [42,43]
	Pam18	DNAJC15	the DnaJ protein Pam18 promotes the formation of a stable mtHsp70–Tim44 complex and stimulates the ATPase activity of mtHsp70 [44–46]
		DNAJC19	
	mtHsp70 (Ssc1)	mtHsp70 (mortalin/ GRP75)	mtHsp70 drives the protein translocation into mitochondrial matrix in an ATP-dependent manner [47]
	Mge1	GRPEL1	Mge1 functions as the nucleotide exchange factor to regulate the mtHsp70 ATPase cycle [48]
		GRPEL2	
mitochondrial chaperonin	Hsp60	HSP60/ HSPD1	mitochondrial chaperonin complex is required for protein folding and complex assembly in the matrix [49,50]
	Hsp10	HSP10/ HSPE1	
matrix-facing AAA protease (mAAA)	Yta10 and Yta12	AFG3L2 and SPG7	the mAAA proteases are IMM-bound, operating as hetero-hexamer of Yta10 and Yta12 in yeast [51] and homo-hexamer of AFG3L2 or hetero-hexamer of AFG3L2 and SPG7 in human [52] to degrade and process proteins on the matrix side
intermembrane space (IMS)-facing AAA protease (iAAA)	Yme1	YME1L1	the iAAA protease is anchored at the IMM and functions as a homo-hexamer with its catalytic domains facing the IMS [53]
long filamentous phenotype (Lon) protease	Pim1	LON1P	the Lon protease functions as a homo-hexamer containing both the ATPase and protease domains, having essential roles in proteostasis in the matrix [54–56]
caseinolytic protease (CLP)	Mcx1	CLPX and CLPP	the human CLP protease system, termed CLPXP, consists of CLPX hexamer to unfold and translocate protein substrate to the double-heptamer CLP protease for degradation [57,58]; yeast mitochondria only contain a non-proteolytic CLPX homologue, Mcx1 [59]
mitochondrial inner membrane insertase	Oxa1	OXA1L	OXA insertase interacts with mitoribosome and facilitates the insertion of newly synthesized mtDNA-encoded proteins into the IMM [60]
mitochondrial ribosome-associated quality control (mitoRQC)	Rqc2	NEMF	Rqc2 and NEMF recognize the 60S–peptidyl-tRNA complex, recruit the E3 ubiquitin ligase Ltn1 and mediate C-terminal tailing to the stalled nascent chain [61–63]
	Ltn1	LTN1	the Ltn1 E3 ligase polyubiquitylates the stalled nascent chain at the 60S ribosomal subunit [64]
	Vms1	ANKZF1	Vms1 endonuclease releases the nascent peptide by tRNA cleavage and counteracts Rqc2 in C-terminal tailing [65,66]
	Pth2	PTRH1	Pth2 is a peptidyl-tRNA hydrolase that releases CAT-tailed proteins arrested at the TOM complex in mitoRQC [67,68]
	Rqc1	TCF25	Rqc1, together with Ltn1-dependent ubiquitylation, recruits the Cdc48–Ufd1–Npl4 complex to extract stalled nascent chain [69]
mitochondrial protein translocation-associated degradation (mitoTAD)	Ubx2	FAF2/UBXD8	Ubx2 and UBXD8 serve as the adaptors to recruit Cdc48 and VCP to the OMM [70,71]; UBXD8 enhances VCP ATPase activity [71]
	Cdc48	VCP/p97	Cdc48 recognizes ubiquitylated, translocation-arrested precursors, using its AAA-ATPase activity to unfold and extract them from the TOM complex for proteasomal degradation [70]
	Ufd1	UFD1L	Ufd1–Npl4 heterodimer functions as a cofactor for Cdc48 [72]
	Npl4	NPLOC4	
mitochondrial compromised protein import response (mitoCPR)	Msp1	ATAD1	Msp1 and ATAD1 are cytosolic AAA-ATPases that remove translocation-arrested precursors from the TOM complex during mitochondrial protein import stress [73,74]
mitophagy	–	PINK1	import-arrested PINK1 kinase is stabilized at the OMM, phosphorylating ubiquitin attached to OMM proteins and the E3 ligase Parkin, resulting in the recruitment of Parkin and its activation at the OMM [75,76]
	Rbd1/Pcp1	PARL	in healthy mitochondria, PARL protease cleaves PINK that is imported to the IMM, releasing it for rapid proteasomal degradation [77]
	–	Parkin	In a feed-forward loop, the activated E3 ligase Parkin ubiquitylates OMM proteins, which are phosphorylated by PINK, recruiting more Parkin. Eventually, ubiquitin chains on mitochondria are recognized by autophagic adapters, leading to mitophagy [78].
the integrated stress response (ISR)	Gcn4	ATF4	the stress-responsive transcription factor ATF4 contains upstream open reading frames (uORFs), mediating its selective translation in the ISR [79]
	–	CHOP	CHOP transcription factor is induced by ATF4 at the transcription level and selectively translated through the regulation of uORF in the ISR [80,81]
	–	DELE1	in mitochondrial stress, import of DELE1 is impaired; the C-terminal fragments of DELE1 are released from protease cleavage, activating the ISR through the HRI pathway [82–84]
	Gcn2	HRI	HRI is one of the kinases that activates the ISR by phosphorylating eIF2α, which suppresses global cap-dependent translation but selectively expresses certain stress-responsive genes [85]
	Oma1	OMA1	OMA1 peptidase is activated during mitochondrial stress, causing DELE1 cleavage and release of a C-terminal fragment, DELE1-S (S for short), to the cytosol to activate HRI [82,83]
	–	HtrA2	mitochondrial protease HtrA2 mediates DELE1 cleavage into DELE1-VS (VS for short) when DELE1 translocation is impaired [84]

functional group	*C. elegans*	human	molecular functions
the mitochondrial unfolded protein response (mtUPR)	ATFS-1	ATF5	the MTS- and NLS-containing transcription factor ATFS-1 is the master regulator of mtUPR in *C. elegans*; ATFS-1 is degraded once imported into mitochondrial matrix but translocated into the nucleus when its mitochondrial import is impaired, which activates the mtUPR [86]
	DNJ-12	DNAJA1	DNAJA1 is a cochaperone of cytosolic HSP70; oxidation of DNAJA1 upon mitochondrial stress enhances the interaction of HSP70 with mitochondrial precursors [87]
	HSP-1	HSP70	during mitochondrial stress, HSP70 is titrated by mitochondrial precursors that are accumulated in the cytosol, releasing HSF1 from HSP70-mediated inhibition to activate the mtUPR [87]
	HSF-1	HSF1	HSF1 transcription factor drives the cellular heat shock response and contributes to the mtUPR in humans in addition to the ATFS-1 orthologue, ATF5 [87]

## Data Availability

This article has no additional data.

## Theme

One contribution of 20 to a Theo Murphy meeting issue ‘ProteostaSys: a systems view of proteostasis’.

## References

[1] Nunnari J , Suomalainen A. 2012 Mitochondria: in sickness and in health. Cell 148, 1145–1159. (10.1016/j.cell.2012.02.035) PMC5381524 22424226

[2] Pfanner N , den Brave F, Becker T. 2025 Mitochondrial protein import stress. Nat. Cell Biol. 27, 188–201. (10.1038/s41556-024-01590-w) 39843636

[3] Ghifari AS , Vazquez-Calvo C, Carlström A, Ott M. 2025 Interconnectivity of mitochondrial protein biogenesis and quality control. Trends Biochem. Sci. 50, 1102–1117. (10.1016/j.tibs.2025.09.004) 41044023

[4] Wiedemann N , Pfanner N. 2017 Mitochondrial machineries for protein import and assembly. Annu. Rev. Biochem. 86, 685–714. (10.1146/annurev-biochem-060815-014352) 28301740

[5] Morgenstern M *et al.* 2021 Quantitative high-confidence human mitochondrial proteome and its dynamics in cellular context. Cell Metab. 33, 2464–2483.e18. (10.1016/j.cmet.2021.11.001) PMC8664129 34800366

[6] Morgenstern M *et al.* 2017 Definition of a high-confidence mitochondrial proteome at quantitative scale. Cell Rep. 19, 2836–2852. (10.1016/j.celrep.2017.06.014) PMC5494306 28658629

[7] Wang X , Chen XJ. 2015 A cytosolic network suppressing mitochondria-mediated proteostatic stress and cell death. Nature 524, 481–484. (10.1038/nature14859) PMC4582408 26192197

[8] Wrobel L *et al.* 2015 Mistargeted mitochondrial proteins activate a proteostatic response in the cytosol. Nature 524, 485–488. (10.1038/nature14951) 26245374

[9] Coyne LP , Wang X, Song J, de Jong E, Schneider K, Massa PT, Middleton FA, Becker T, Chen XJ. 2023 Mitochondrial protein import clogging as a mechanism of disease. eLife 12, e84330. (10.7554/eLife.84330) PMC10208645 37129366

[10] Wang X , Middleton FA, Tawil R, Chen XJ. 2022 Cytosolic adaptation to mitochondria-induced proteostatic stress causes progressive muscle wasting. iScience 25, 103715. (10.1016/j.isci.2021.103715) PMC8762400 35072007

[11] Doan KN . 2020 The mitochondrial import complex MIM functions as main translocase for α-helical outer membrane proteins. Cell Rep. 31, 107567. (10.1016/j.celrep.2020.107567) 32348752

[12] Takeda H . 2021 Mitochondrial sorting and assembly machinery operates by β-barrel switching. Nature 590, 163–169. (10.1038/s41586-020-03113-7) 33408415

[13] Rehling P *et al.* 2003 Protein insertion into the mitochondrial inner membrane by a twin-pore translocase. Science 299, 1747–1751. (10.1126/science.1080945) 12637749

[14] Koch, JR, Schmid, FX. 2014 Mia40 targets cysteines in a hydrophobic environment to direct oxidative protein folding in the mitochondria. Nat. Commun. 5, 3041. (10.1038/ncomms4041) 24407114

[15] Vögtle F-N *et al.* 2009 Global analysis of the mitochondrial N-proteome identifies a processing peptidase critical for protein stability. Cell 139, 428–439. (10.1016/j.cell.2009.07.045) 19837041

[16] Bykov, YS, Flohr, T, Boos, F, Zung, N, Herrmann, JM, Schuldiner, M. 2022 Widespread use of unconventional targeting signals in mitochondrial ribosome proteins. EMBO J. 41, EMBJ2021109519. (10.15252/embj.2021109519) PMC8724765 34786732

[17] Sim, SI, Chen, Y, Lynch, DL, Gumbart, JC, Park, E. 2023 Structural basis of mitochondrial protein import by the TIM23 complex. Nature 621, 620–626. (10.1038/s41586-023-06239-6) PMC11495887 37344598

[18] Fielden LF *et al.* 2023 Central role of Tim17 in mitochondrial presequence protein translocation. Nature 621, 627–634. (10.1038/s41586-023-06477-8) PMC10511324 37527780

[19] Mordas A , Tokatlidis K. 2015 The MIA pathway: a key regulator of mitochondrial oxidative protein folding and biogenesis. Acc. Chem. Res. 48, 2191–2199. (10.1021/acs.accounts.5b00150) PMC4551283 26214018

[20] Eaglesfield R , Tokatlidis K. 2021 Targeting and insertion of membrane proteins in mitochondria. Front. Cell Dev. Biol. 9, 803205. (10.3389/fcell.2021.803205) PMC8740019 35004695

[21] Tucker K , Park E. 2019 Cryo-EM structure of the mitochondrial protein-import channel TOM complex at near-atomic resolution. Nat. Struct. Mol. Biol. 26, 1158–1166. (10.1038/s41594-019-0339-2) PMC8439582 31740857

[22] Araiso Y *et al.* 2019 Structure of the mitochondrial import gate reveals distinct preprotein paths. Nature 575, 395–401. (10.1038/s41586-019-1680-7) 31600774

[23] Bausewein T , Mills DJ, Langer JD, Nitschke B, Nussberger S, Kühlbrandt W. 2017 Cryo-EM structure of the TOM core complex from *Neurospora crassa*. Cell 170, 693–700.e7. (10.1016/j.cell.2017.07.012) 28802041

[24] Dietmeier K , Hönlinger A, Bömer U, Dekker PJT, Eckerskorn C, Lottspeich F, Kübrich M, Pfanner N. 1997 Tom5 functionally links mitochondrial preprotein receptors to the general import pore. Nature 388, 195–200. (10.1038/40663) 9217162

[25] Hönlinger A , Bömer U, Alconada A, Eckerskorn C, Lottspeich F, Dietmeier K, Pfanner N. 1996 Tom7 modulates the dynamics of the mitochondrial outer membrane translocase and plays a pathway-related role in protein import. EMBO J. 15, 2125–2137. (10.1002/j.1460-2075.1996.tb00566.x) PMC450135 8641278

[26] Abe Y , Shodai T, Muto T, Mihara K, Torii H, Nishikawa S-i, Endo T, Kohda D. 2000 Structural basis of presequence recognition by the mitochondrial protein import receptor Tom20. Cell 100, 551–560. (10.1016/s0092-8674(00)80691-1) 10721992

[27] Moczko M , Bömer U, Kübrich M, Zufall N, Hönlinger A, Pfanner N. 1997 The intermembrane space domain of mitochondrial Tom22 functions as a* trans* binding site for preproteins with N-terminal targeting sequences. Mol. Cell. Biol. 17, 6574–6584. (10.1128/mcb.17.11.6574) PMC232511 9343421

[28] Rapaport D , Künkele KP, Dembowski M, Ahting U, Nargang FE, Neupert W, Lill R. 1998 Dynamics of the TOM complex of mitochondria during binding and translocation of preproteins. Mol. Cell. Biol. 18, 5256–5262. (10.1128/mcb.18.9.5256) PMC109111 9710610

[29] Backes S *et al.* 2021 The chaperone-binding activity of the mitochondrial surface receptor Tom70 protects the cytosol against mitoprotein-induced stress. Cell Rep. 35, 108936. (10.1016/j.celrep.2021.108936) PMC7615001 33826901

[30] Yamamoto H *et al.* 2009 Roles of Tom70 in import of presequence-containing mitochondrial proteins. J. Biol. Chem. 284, 31635–31646. (10.1074/jbc.M109.041756). PMC2797234 19767391

[31] Sinha D , Srivastava S, Krishna L, D'Silva P. 2014 Unraveling the intricate organization of mammalian mitochondrial presequence translocases: existence of multiple translocases for maintenance of mitochondrial function. Mol. Cell. Biol. 34, 1757–1775. (10.1128/MCB.01527-13) PMC4019027 24636990

[32] Hsu MC , Kinefuchi H, Lei L, Kikuchi R, Yamano K, Youle RJ. 2025 Mitochondrial YME1L1 governs unoccupied protein translocase channels. Nat. Cell Biol. 27, 309–321. (10.1038/s41556-024-01571-z) 39774271

[33] Rainbolt TK , Atanassova N, Genereux JC, Wiseman RL. 2013 Stress-regulated translational attenuation adapts mitochondrial protein import through Tim17A degradation. Cell Metab. 18, 908–919. (10.1016/j.cmet.2013.11.006) PMC3904643 24315374

[34] MacVicar T *et al.* 2019 Lipid signalling drives proteolytic rewiring of mitochondria by YME1L. Nature 575, 361–365. (10.1038/s41586-019-1738-6) 31695197

[35] Nguyen NGT . 2025 HSF1 remodels mitochondrial biogenesis and function in cancer cells via TIMM17A. Biorxiv. (10.1101/2025.05.12.653547)

[36] Yamamoto H , Esaki M, Kanamori T, Tamura Y, Nishikawa S-i, Endo T. 2002 Tim50 is a subunit of the TIM23 complex that links protein translocation across the outer and inner mitochondrial membranes. Cell 111, 519–528. (10.1016/s0092-8674(02)01053-x) 12437925

[37] Qian X , Gebert M, Höpker J, Yan M, Li J, Wiedemann N, van der Laan M, Pfanner N, Sha B. 2011 Structural basis for the function of Tim50 in the mitochondrial presequence translocase. J. Mol. Biol. 411, 513–519. (10.1016/j.jmb.2011.06.020) PMC3146634 21704637

[38] Geissler A . 2002 The mitochondrial presequence translocase: an essential role of Tim50 in directing preproteins to the import channel. Cell 111, 507–518. (10.1016/S0092-8674(02)01073-5) 12437924

[39] Richter F *et al.* 2019 ROMO1 is a constituent of the human presequence translocase required for YME1L protease import. J. Cell Biol. 218, 598–614. (10.1083/jcb.201806093) PMC6363466 30598479

[40] Ieva R *et al.* 2014 Mgr2 functions as lateral gatekeeper for preprotein sorting in the mitochondrial inner membrane. Mol. Cell 56, 641–652. (10.1016/j.molcel.2014.10.010) 25454944

[41] D'Silva, PR, Schilke, B, Hayashi, M, Craig, EA. 2008 Interaction of the J-protein heterodimer Pam18/Pam16 of the mitochondrial import motor with the translocon of the inner membrane. Mol. Biol. Cell 19, 424–432. (10.1091/mbc.e07-08-0748) PMC2174176 18003975

[42] Li Y , Dudek J, Guiard B, Pfanner N, Rehling P, Voos W. 2004 The presequence translocase-associated protein import motor of mitochondria: Pam16 functions in an antagonist manner to Pam18. J. Biol. Chem. 279, 38047–38054.(10.1074/jbc.M404319200) 15218029

[43] Frazier AE *et al.* 2004 Pam16 has an essential role in the mitochondrial protein import motor. Nat. Struct. Mol. Biol. 11, 226–233. (10.1038/nsmb735) 14981507

[44] Truscott KN . 2003 A J-protein is an essential subunit of the presequence translocase-associated protein import motor of mitochondria. J. Cell Biol. 163, 707–713. (10.1083/jcb.200308004) PMC2173675 14638855

[45] Mokranjac D , Sichting M, Neupert W, Hell K. 2003 Tim14, a novel key component of the import motor of the TIM23 protein translocase of mitochondria. EMBO J. 22, 4945–4956. (10.1093/emboj/cdg485) PMC204468 14517234

[46] D'Silva PD , Schilke B, Walter W, Andrew A, Craig EA. 2003 J protein cochaperone of the mitochondrial inner membrane required for protein import into the mitochondrial matrix. Proc. Natl Acad. Sci. USA 100, 13839–13844. (10.1073/pnas.1936150100) PMC283508 14605210

[47] Schneider H-C , Berthold J, Bauer MF, Dietmeier K, Guiard B, Brunner M, Neupert W. 1994 Mitochondrial Hsp70/MIM44 complex facilitates protein import. Nature 371, 768–774. (10.1038/371768a0) 7935837

[48] Schneider HC , Westermann B, Neupert W, Brunner M. 1996 The nucleotide exchange factor MGE exerts a key function in the ATP-dependent cycle of mt-Hsp70-Tim44 interaction driving mitochondrial protein import. EMBO J. 15, 5796–5803. (10.1002/j.1460-2075.1996.tb00966.x) PMC452327 8918457

[49] Ostermann J , Horwich AL, Neupert W, Hartl FU. 1989 Protein folding in mitochondria requires complex formation with hsp60 and ATP hydrolysis. Nature 341, 125–130. (10.1038/341125a0) 2528694

[50] Cheng MY , Hartl F-U, Martin J, Pollock RA, Kalousek F, Neuper W, Hallberg EM, Hallberg RL, Horwich AL. 1989 Mitochondrial heat-shock protein hsp60 is essential for assembly of proteins imported into yeast mitochondria. Nature 337, 620–625. (10.1038/337620a0) 2645524

[51] Arlt H , Tauer R, Feldmann H, Neupert W, Langer T. 1996 The YTA10–12 complex, an AAA protease with chaperone-like activity in the inner membrane of mitochondria. Cell 85, 875–885. (10.1016/s0092-8674(00)81271-4) 8681382

[52] Koppen M , Metodiev MD, Casari G, Rugarli EI, Langer T. 2007 Variable and tissue-specific subunit composition of mitochondrial m-AAA protease complexes linked to hereditary spastic paraplegia. Mol. Cell. Biol. 27, 758–767. (10.1128/MCB.01470-06) PMC1800790 17101804

[53] Puchades C , Rampello AJ, Shin M, Giuliano CJ, Wiseman RL, Glynn SE, Lander GC. 2017 Structure of the mitochondrial inner membrane AAA+ protease YME1 gives insight into substrate processing. Science 358, eaao0464. (10.1126/science.aao0464) PMC5829300 29097521

[54] Suzuki CK , Suda K, Wang N, Schatz G. 1994 Requirement for the yeast gene *LON* in intramitochondrial proteolysis and maintenance of respiration. Science 264, 273–276. (10.1126/science.8146662) 8146662

[55] Shin M , Watson ER, Song AS, Mindrebo JT, Novick SJ, Griffin PR, Wiseman RL, Lander GC. 2021 Structures of the human LONP1 protease reveal regulatory steps involved in protease activation. Nat. Commun. 12, 3239. (10.1038/s41467-021-23495-0) PMC8163871 34050165

[56] Shin CS , Meng S, Garbis SD, Moradian A, Taylor RW, Sweredoski MJ, Lomenick B, Chan DC. 2021 LONP1 and mtHSP70 cooperate to promote mitochondrial protein folding. Nat. Commun. 12, 265. (10.1038/s41467-020-20597-z) PMC7801493 33431889

[57] Kang SG , Ortega J, Singh SK, Wang N, Huang N-n, Steven AC, Maurizi MR. 2002 Functional proteolytic complexes of the human mitochondrial ATP-dependent protease, hClpXP. J. Biol. Chem. 277, 21095–21102. (10.1074/jbc.M201642200) 11923310

[58] Gatsogiannis C , Balogh D, Merino F, Sieber SA, Raunser S. 2019 Cryo-EM structure of the ClpXP protein degradation machinery. Nat. Struct. Mol. Biol. 26, 946–954. (10.1038/s41594-019-0304-0) PMC6783313 31582852

[59] Kardon JR , Moroco JA, Engen JR, Baker TA. 2020 Mitochondrial ClpX activates an essential biosynthetic enzyme through partial unfolding. eLife 9, e54387. (10.7554/eLife.54387) PMC7077987 32091391

[60] Homberg B , Rehling P, Cruz-Zaragoza LD. 2023 The multifaceted mitochondrial OXA insertase. Trends Cell Biol. 33, 765–772. (10.1016/j.tcb.2023.02.001) 36863885

[61] Filbeck S , Cerullo F, Pfeffer S, Joazeiro CAP. 2022 Ribosome-associated quality-control mechanisms from bacteria to humans. Mol. Cell 82, 1451–1466. (10.1016/j.molcel.2022.03.038) PMC9034055 35452614

[62] Kostova KK , Hickey KL, Osuna BA, Hussmann JA, Frost A, Weinberg DE, Weissman JS. 2017 CAT-tailing as a fail-safe mechanism for efficient degradation of stalled nascent polypeptides. Science 357, 414–417. (10.1126/science.aam7787) PMC5673106 28751611

[63] Thrun A *et al.* 2021 Convergence of mammalian RQC and C-end rule proteolytic pathways via alanine tailing. Mol. Cell 81, 2112–2122.e7. (10.1016/j.molcel.2021.03.004) PMC8141035 33909987

[64] Brandman O *et al.* 2012 A ribosome-bound quality control complex triggers degradation of nascent peptides and signals translation stress. Cell 151, 1042–1054. (10.1016/j.cell.2012.10.044) PMC3534965 23178123

[65] Izawa T , Park SH, Zhao L, Hartl FU, Neupert W. 2017 Cytosolic protein Vms1 links ribosome quality control to mitochondrial and cellular homeostasis. Cell 171, 890–903.e18. (10.1016/j.cell.2017.10.002) 29107329

[66] Verma R , Reichermeier KM, Burroughs AM, Oania RS, Reitsma JM, Aravind L, Deshaies RJ. 2018 Vms1 and ANKZF1 peptidyl-tRNA hydrolases release nascent chains from stalled ribosomes. Nature 557, 446–451. (10.1038/s41586-018-0022-5) PMC6226276 29632312

[67] Schulte U *et al.* 2023 Mitochondrial complexome reveals quality-control pathways of protein import. Nature 614, 153–159. (10.1038/s41586-022-05641-w) PMC9892010 36697829

[68] Bertram N *et al.* 2025 Delayed protein translocation protects mitochondria against toxic CAT-tailed proteins. Mol. Cell 85, 4082–4092.e7. (10.1016/j.molcel.2025.09.030) 41118763

[69] Defenouillère Q *et al.* 2013 Cdc48-associated complex bound to 60S particles is required for the clearance of aberrant translation products. Proc. Natl Acad. Sci. USA 110, 5046–5051. (10.1073/pnas.1221724110) PMC3612664 23479637

[70] Mårtensson CU *et al.* 2019 Mitochondrial protein translocation-associated degradation. Nature 569, 679–683. (10.1038/s41586-019-1227-y) 31118508

[71] Huo XY , Liu D, Zou R, Li ZP, Li Y, Pan L, Zhang Y, Zhang ZR. 2025 A UBH-UBX module amplifies p97/VCP's unfolding power to facilitate protein extraction and degradation. Nat. Commun. 16, 10162. (10.1038/s41467-025-65166-4) PMC12630741 41258083

[72] Twomey EC , Ji Z, Wales TE, Bodnar NO, Ficarro SB, Marto JA, Engen JR, Rapoport TA. 2019 Substrate processing by the Cdc48 ATPase complex is initiated by ubiquitin unfolding. Science 365, eaax1033. (10.1126/science.aax1033) PMC6980381 31249135

[73] Weidberg H , Amon A. 2018 MitoCPR-A surveillance pathway that protects mitochondria in response to protein import stress. Science 360, eaan4146. (10.1126/science.aan4146) PMC6528467 29650645

[74] Kim J , Goldstein M, Zecchel L, Ghorayeb R, Maxwell CA, Weidberg H. 2024 ATAD1 prevents clogging of TOM and damage caused by un-imported mitochondrial proteins. Cell Rep. 43, 114473. (10.1016/j.celrep.2024.114473) 39024102

[75] Wauer T , Simicek M, Schubert A, Komander D. 2015 Mechanism of phospho-ubiquitin-induced PARKIN activation. Nature 524, 370–374. (10.1038/nature14879) PMC4984986 26161729

[76] Kazlauskaite A *et al.* 2015 Binding to serine 65-phosphorylated ubiquitin primes Parkin for optimal PINK1-dependent phosphorylation and activation. EMBO Rep. 16, 939–954. (10.15252/embr.201540352) PMC4552487 26116755

[77] Jin SM , Lazarou M, Wang C, Kane LA, Narendra DP, Youle RJ. 2010 Mitochondrial membrane potential regulates PINK1 import and proteolytic destabilization by PARL. J. Cell Biol. 191, 933–942. (10.1083/jcb.201008084) PMC2995166 21115803

[78] Wang S *et al.* 2023 The mitophagy pathway and its implications in human diseases. Signal. Transduct. Target Ther. 8, 304. (10.1038/s41392-023-01503-7) PMC10427715 37582956

[79] Vattem KM , Wek RC. 2004 Reinitiation involving upstream ORFs regulates ATF4 mRNA translation in mammalian cells. Proc. Natl Acad. Sci. USA 101, 11269–11274. (10.1073/pnas.0400541101) PMC509193 15277680

[80] Harding HP , Novoa I, Zhang Y, Zeng H, Wek R, Schapira M, Ron D. 2000 Regulated translation initiation controls stress-induced gene expression in mammalian cells. Mol. Cell 6, 1099–1108. (10.1016/s1097-2765(00)00108-8) 11106749

[81] Palam LR , Baird TD, Wek RC. 2011 Phosphorylation of eIF2 facilitates ribosomal bypass of an inhibitory upstream ORF to enhance CHOP translation. J. Biol. Chem. 286, 10939–10949. (10.1074/jbc.M110.216093) PMC3064149 21285359

[82] Fessler E *et al.* 2020 A pathway coordinated by DELE1 relays mitochondrial stress to the cytosol. Nature 579, 433–437. (10.1038/s41586-020-2076-4) PMC7116715 32132706

[83] Guo X . 2020 Mitochondrial stress is relayed to the cytosol by an OMA1–DELE1–HRI pathway. Nature 579, 427–432. (10.1038/s41586-020-2078-2) PMC7147832 32132707

[84] Bi PY , Killackey SA, Schweizer L, Arnoult D, Philpott DJ, Girardin SE. 2024 Cytosolic retention of HtrA2 during mitochondrial protein import stress triggers the DELE1-HRI pathway. Commun. Biol. 7, 391. (10.1038/s42003-024-06107-7) PMC10981713 38555279

[85] Chen JJ . 2025 HRI protein kinase in cytoplasmic heme sensing and mitochondrial stress response: relevance to hematological and mitochondrial diseases. J. Biol. Chem. 301, 108494. (10.1016/j.jbc.2025.108494) PMC12127572 40209956

[86] Nargund AM , Pellegrino MW, Fiorese CJ, Baker BM, Haynes CM. 2012 Mitochondrial import efficiency of ATFS-1 regulates mitochondrial UPR activation. Science 337, 587–590. (10.1126/science.1223560) PMC3518298 22700657

[87] Sutandy FXR , Gößner I, Tascher G, Münch C. 2023 A cytosolic surveillance mechanism activates the mitochondrial UPR. Nature 618, 849–854. (10.1038/s41586-023-06142-0) PMC10284689 37286597

[88] Yamano K , Yatsukawa Y, Esaki M, Hobbs AE, Jensen RE, Endo T. 2008 Tom20 and Tom22 share the common signal recognition pathway in mitochondrial protein import. J. Biol. Chem. 283, 3799–3807. (10.1074/jbc.M708339200) 18063580

[89] Srivastava S , Savanur MA, Sinha D, Birje A, R V, Saha PP, D'Silva, P. 2017 Regulation of mitochondrial protein import by the nucleotide exchange factors GrpEL1 and GrpEL2 in human cells. J. Biol. Chem. 292, 18075–18090. (10.1074/jbc.M117.788463) PMC5672033 28848044

[90] Zhou X , Yang Y, Wang G, Wang S, Sun D, Ou X, Lian Y, Li L. 2023 Molecular pathway of mitochondrial preprotein import through the TOM–TIM23 supercomplex. Nat. Struct. Mol. Biol. 30, 1996–2008. (10.1038/s41594-023-01103-7) 37696957

[91] Vazquez‐Calvo C , Kohler V, Höög JL, Büttner S, Ott M. 2023 Newly imported proteins in mitochondria are particularly sensitive to aggregation. Acta Physiol. 238, e13985. (10.1111/apha.13985) PMC10909475 37171464

[92] Itoh Y , Andréll J, Choi A, Richter U, Maiti P, Best RB, Barrientos A, Battersby BJ, Amunts A. 2021 Mechanism of membrane-tethered mitochondrial protein synthesis. Science 371, 846–849. (10.1126/science.abe0763) PMC7610362 33602856

[93] Singh AP , Salvatori R, Aftab W, Kohler A, Carlström A, Forne I, Imhof A, Ott M. 2020 Molecular connectivity of mitochondrial gene expression and OXPHOS biogenesis. Mol. Cell 79, 1051–1065.e10. (10.1016/j.molcel.2020.07.024) 32877643

[94] Kohler A *et al.* 2023 Early fate decision for mitochondrially encoded proteins by a molecular triage. Mol. Cell 83, 3470–3484.e8. (10.1016/j.molcel.2023.09.001) 37751741

[95] Steglich G , Neupert W, Langer T. 1999 Prohibitins regulate membrane protein degradation by the m-AAA protease in mitochondria. Mol. Cell. Biol. 19, 3435–3442. (10.1128/MCB.19.5.3435) PMC84136 10207067

[96] Lange F , Ratz M, Dohrke JN, Le Vasseur M, Wenzel D, Ilgen P, Riedel D, Jakobs S. 2025 In situ architecture of the human prohibitin complex. Nat. Cell Biol. 27, 633–640. (10.1038/s41556-025-01620-1) PMC11991916 40119201

[97] Hong S , Guan Z, Zhang L, Zhuang J, Yan L, Liu Y, Liu Z, Wang Q, Yin P. 2025 Cryo-EM structure of the prohibitin complex in open conformation. Proc. Natl Acad. Sci. USA 122, e2512430122. (10.1073/pnas.2512430122) PMC12478178 40966277

[98] Luo D , Zheng L, Lu MA, Chen Z, Wang P, Guo Q, Gao N. 2025 Structural basis for prohibitin-mediated regulation of mitochondrial m-AAA protease. Nat. Commun. 17, 635. (10.1101/2025.04.15.648916). PMC13376388 42129162

[99] Shpilka T . 2021 UPR^mt^ scales mitochondrial network expansion with protein synthesis via mitochondrial import in *Caenorhabditis elegans*. Nat. Commun. 12, 479. (10.1038/s41467-020-20784-y) PMC7817664 33473112

[100] Melber A , Haynes CM. 2018 UPR^mt^ regulation and output: a stress response mediated by mitochondrial-nuclear communication. Cell Res. 28, 281–295. (10.1038/cr.2018.16) PMC5835775 29424373

[101] Perez-Martinez X , Butler CA, Shingu-Vazquez M, Fox TD. 2009 Dual functions of Mss51 couple synthesis of Cox1 to assembly of cytochrome *c* oxidase in *Saccharomyces cerevisiae* mitochondria. Mol. Biol. Cell 20, 4371–4380. (10.1091/mbc.e09-06-0522) PMC2762147 19710419

[102] Gruschke S , Römpler K, Hildenbeutel M, Kehrein K, Kühl I, Bonnefoy N, Ott M. 2012 The Cbp3–Cbp6 complex coordinates cytochrome b synthesis with *bc*_*1*_ complex assembly in yeast mitochondria. J. Cell Biol. 199, 137–150. (10.1083/jcb.201206040) PMC3461508 23007649

[103] Seshadri SR , Banarjee C, Barros MH, Fontanesi F. 2020 The translational activator Sov1 coordinates mitochondrial gene expression with mitoribosome biogenesis. Nucleic Acids Res. 48, 6759–6774. (10.1093/nar/gkaa424) PMC7337963 32449921

[104] Kabala AM *et al.* 2022 Assembly-dependent translation of subunits 6 (Atp6) and 9 (Atp9) of ATP synthase in yeast mitochondria. Genetics 220, iyac007. (10.1093/genetics/iyac007) PMC8893259 35100419

[105] Salvatori R , Kehrein K, Singh AP, Aftab W, Möller-Hergt BV, Forne I, Imhof A, Ott M. 2020 Molecular wiring of a mitochondrial translational feedback loop. Mol. Cell 77, 887–900.e5. (10.1016/j.molcel.2019.11.019) 31883951

[106] Carlström A , Bridgers JB, Couvillion M, Singh AP, Forné I, Imhof A, Churchman LS, Ott M. 2025 A molecular switch at the yeast mitoribosomal tunnel exit controls cytochrome *b* synthesis. Nucleic Acids Res. 53, gkaf634. (10.1093/nar/gkaf634) PMC12242767 40637229

[107] Couvillion MT , Soto IC, Shipkovenska G, Churchman LS. 2016 Synchronized mitochondrial and cytosolic translation programs. Nature 533, 499–503. (10.1038/nature18015) PMC4964289 27225121

[108] Richter-Dennerlein R *et al.* 2016 Mitochondrial protein synthesis adapts to influx of nuclear-encoded protein. Cell 167, 471–483.e10. (10.1016/j.cell.2016.09.003) PMC5055049 27693358

[109] Maruszczak KK , Draczkowski P, Wnorowski A, Chacinska A. 2024 Structure prediction analysis of human core TIM23 complex reveals conservation of the protein translocation mechanism. FEBS Open Bio 14, 1656–1667. (10.1002/2211-5463.13840) PMC11452300 38837610

[110] Elancheliyan P *et al.* 2024 OCIAD1 and prohibitins regulate the stability of the TIM23 protein translocase. Cell Rep. 43, 115038. (10.1016/j.celrep.2024.115038) PMC11672691 39630581

[111] Kroczek L , Nolte H, Lasarzewski Y, Agrawal I, Molinié T, Curbelo Piñero D, Lemke K, Rugarli E, Langer T. 2026 Stress adaptation of mitochondrial protein import by OMA1-mediated degradation of DNAJC15. Nat. Struct. Mol. Biol. 33, 499–511. (10.1101/2025.03.04.641455) PMC12999506 41760807

[112] Konovalova S *et al.* 2018 Redox regulation of GRPEL2 nucleotide exchange factor for mitochondrial HSP70 chaperone. Redox Biol. 19, 37–45. (10.1016/j.redox.2018.07.024) PMC6089081 30098457

[113] Manjunath P , Stojkovič G, Euro L, Konovalova S, Wanrooij S, Koski K, Tyynismaa H. 2024 Preferential binding of ADP-bound mitochondrial HSP70 to the nucleotide exchange factor GRPEL1 over GRPEL2. Protein Sci. 33, e5190. (10.1002/pro.5190) PMC11500471 39445986

[114] Miao L , Wu D, Zhao H, Xie A. 2024 TIMM17A overexpression in lung adenocarcinoma and its association with prognosis. Scient. Rep. 14, 8840. (10.1038/s41598-024-59526-1) PMC11024209 38632467

[115] Sinha D , Srivastava S, D'Silva P. 2016 Functional diversity of human mitochondrial J-proteins is independent of their association with the inner membrane presequence translocase. J. Biol. Chem. 291, 17345–17359. (10.1074/jbc.M116.738146) PMC5016132 27330077

[116] Brodsky JL . 2012 Cleaning up: ER-associated degradation to the rescue. Cell 151, 1163–1167. (10.1016/j.cell.2012.11.012) PMC3521611 23217703

[117] Phu L , Rose CM, Tea JS, Wall CE, Verschueren E, Cheung TK, Kirkpatrick DS, Bingol B. 2020 Dynamic regulation of mitochondrial import by the ubiquitin system. Mol. Cell 77, 1107–1123.e10. (10.1016/j.molcel.2020.02.012) 32142684

[118] Neuber O , Jarosch E, Volkwein C, Walter J, Sommer T. 2005 Ubx2 links the Cdc48 complex to ER-associated protein degradation. Nat. Cell Biol. 7, 993–998. (10.1038/ncb1298) 16179953

[119] Schuberth C , Buchberger A. 2005 Membrane-bound Ubx2 recruits Cdc48 to ubiquitin ligases and their substrates to ensure efficient ER-associated protein degradation. Nat. Cell Biol. 7, 999–1006. (10.1038/ncb1299) 16179952

[120] Yuan Z . 2026 A direct role for a mitochondrial targeting sequence in signalling stress. Nature 649, 1302–1311. (10.1038/s41586-025-09834-x). PMC7618714 41372412

[121] Williams CC , Jan CH, Weissman JS. 2014 Targeting and plasticity of mitochondrial proteins revealed by proximity-specific ribosome profiling. Science 346, 748–751. (10.1126/science.1257522) PMC4263316 25378625

[122] Joazeiro CAP . 2019 Mechanisms and functions of ribosome-associated protein quality control. Nat. Rev. Mol. Cell Biol. 20, 368–383. (10.1038/s41580-019-0118-2) PMC7138134 30940912

[123] Choe Y-J , Park S-H, Hassemer T, Körner R, Vincenz-Donnelly L, Hayer-Hartl M, Hartl FU. 2016 Failure of RQC machinery causes protein aggregation and proteotoxic stress. Nature 531, 191–195. (10.1038/nature16973) 26934223

[124] Kuroha K , Zinoviev A, Hellen CUT, Pestova TV. 2018 Release of ubiquitinated and non-ubiquitinated nascent chains from stalled mammalian ribosomal complexes by ANKZF1 and Ptrh1. Mol. Cell 72, 286–302.e8. (10.1016/j.molcel.2018.08.022) PMC6344051 30244831

[125] Lv L *et al.* 2024 NEMF-mediated listerin-independent mitochondrial translational surveillance by E3 ligase Pirh2 and mitochondrial protease ClpXP. Cell Rep. 43, 113860. (10.1016/j.celrep.2024.113860) 38412092

[126] Rolland SG . 2019 Compromised mitochondrial protein import acts as a signal for UPR^mt^. Cell Rep. 28, 1659–1669.e5. (10.1016/j.celrep.2019.07.049) 31412237

[127] Haynes CM , Petrova K, Benedetti C, Yang Y, Ron D. 2007 ClpP mediates activation of a mitochondrial unfolded protein response in *C. elegans*. Dev. Cell 13, 467–480. (10.1016/j.devcel.2007.07.016) 17925224

[128] Martinus RD , Garth GP, Webster TL, Cartwright P, Naylor DJ, Høj PB, Hoogenraad NJ. 1996 Selective induction of mitochondrial chaperones in response to loss of the mitochondrial genome. Eur. J. Biochem. 240, 98–103. (10.1111/j.1432-1033.1996.0098h.x) 8797841

[129] Yoneda T , Benedetti C, Urano F, Clark SG, Harding HP, Ron D. 2004 Compartment-specific perturbation of protein handling activates genes encoding mitochondrial chaperones. J. Cell Sci. 117, 4055–4066. (10.1242/jcs.01275) 15280428

[130] Houtkooper RH , Mouchiroud L, Ryu D, Moullan N, Katsyuba E, Knott G, Williams RW, Auwerx J. 2013 Mitonuclear protein imbalance as a conserved longevity mechanism. Nature 497, 451–457. (10.1038/nature12188) PMC3663447 23698443

[131] Münch C , Harper JW. 2016 Mitochondrial unfolded protein response controls matrix pre-RNA processing and translation. Nature 534, 710–713. (10.1038/nature18302) PMC4939261 27350246

[132] Zhao Q , Wang J, Levichkin IV, Stasinopoulos S, Ryan MT, Hoogenraad NJ. 2002 A mitochondrial specific stress response in mammalian cells. EMBO J. 21, 4411–4419. (10.1093/emboj/cdf445) PMC126185 12198143

[133] Nargund AM , Fiorese CJ, Pellegrino MW, Deng P, Haynes CM. 2015 Mitochondrial and nuclear accumulation of the transcription factor ATFS-1 promotes OXPHOS recovery during the UPR^mt^. Mol. Cell 58, 123–133. (10.1016/j.molcel.2015.02.008) PMC4385436 25773600

[134] Xin N , Durieux J, Yang C, Wolff S, Kim HE, Dillin A. 2022 The UPR^mt^ preserves mitochondrial import to extend lifespan. J. Cell Biol. 221, e202201071. (10.1083/jcb.202201071) PMC9134095 35608535

[135] Moehle EA , Shen K, Dillin A. 2019 Mitochondrial proteostasis in the context of cellular and organismal health and aging. J. Biol. Chem. 294, 5396–5407. (10.1074/jbc.TM117.000893) PMC6462515 29622680

[136] Anderson NS , Haynes CM. 2020 Folding the mitochondrial UPR into the integrated stress response. Trends Cell Biol. 30, 428–439. (10.1016/j.tcb.2020.03.001) PMC7230072 32413314

[137] Fiorese CJ , Schulz AM, Lin YF, Rosin N, Pellegrino MW, Haynes CM. 2016 The transcription factor ATF5 mediates a mammalian mitochondrial UPR. Curr. Biol. 26, 2037–2043. (10.1016/j.cub.2016.06.002) PMC4980197 27426517

[138] Li G , Xu Y, Guan D, Liu Z, Liu DX. 2011 HSP70 protein promotes survival of C6 and U87 glioma cells by inhibition of ATF5 degradation. J. Biol. Chem. 286, 20251–20259. (10.1074/jbc.M110.211771) PMC3121475 21521685

[139] Zhou D , Palam LR, Jiang L, Narasimhan J, Staschke KA, Wek RC. 2008 Phosphorylation of eIF2 directs ATF5 translational control in response to diverse stress conditions. J. Biol. Chem. 283, 7064–7073. (10.1074/jbc.M708530200) 18195013

[140] Vihervaara A , Sistonen L. 2014 HSF1 at a glance. J. Cell Sci. 127, 261–266. (10.1242/jcs.132605) 24421309

[141] Sekine Y , Houston R, Eckl EM, Fessler E, Narendra DP, Jae LT, Sekine S. 2023 A mitochondrial iron-responsive pathway regulated by DELE1. Mol. Cell 83, 2059–2076.e6. (10.1016/j.molcel.2023.05.031) PMC10329284 37327776

[142] Kaspar S *et al.* 2021 Adaptation to mitochondrial stress requires CHOP-directed tuning of ISR. Sci. Adv. 7, eabf0971. (10.1126/sciadv.abf0971) PMC8153728 34039602

[143] Narendra DP , Jin SM, Tanaka A, Suen DF, Gautier CA, Shen J, Cookson MR, Youle RJ. 2010 PINK1 is selectively stabilized on impaired mitochondria to activate Parkin. PLoS Biol. 8, e1000298. (10.1371/journal.pbio.1000298) PMC2811155 20126261

[144] Eldeeb MA *et al.* 2024 Tom20 gates PINK1 activity and mediates its tethering of the TOM and TIM23 translocases upon mitochondrial stress. Proc. Natl Acad. Sci. USA 121, e2313540121. (10.1073/pnas.2313540121) PMC10927582 38416681

[145] Hasson SA *et al.* 2013 High-content genome-wide RNAi screens identify regulators of parkin upstream of mitophagy. Nature 504, 291–295. (10.1038/nature12748) PMC5841086 24270810

[146] Akabane S *et al.* 2023 TIM23 facilitates PINK1 activation by safeguarding against OMA1-mediated degradation in damaged mitochondria. Cell Rep. 42, 112454. (10.1016/j.celrep.2023.112454) 37160114

[147] Sekine S , Wang C, Sideris DP, Bunker E, Zhang Z, Youle RJ. 2019 Reciprocal roles of Tom7 and OMA1 during mitochondrial import and activation of PINK1. Mol. Cell 73, 1028–1043.e5. (10.1016/j.molcel.2019.01.002) 30733118

[148] Okatsu K *et al.* 2012 PINK1 autophosphorylation upon membrane potential dissipation is essential for Parkin recruitment to damaged mitochondria. Nat. Commun. 3, 1016. (10.1038/ncomms2016) PMC3432468 22910362

[149] Kondapalli C . 2012 PINK1 is activated by mitochondrial membrane potential depolarization and stimulates Parkin E3 ligase activity by phosphorylating serine 65. Open Biol. 2, 120080. (10.1098/rsob.120080) PMC3376738 22724072

[150] Sauvé V . 2015 A Ubl/ubiquitin switch in the activation of Parkin. EMBO J. 34, 2492–2505. (10.15252/embj.201592237) PMC4609182 26254305

[151] Michaelis JB , Brunstein ME, Bozkurt S, Alves L, Wegner M, Kaulich M, Pohl C, Münch C. 2022 Protein import motor complex reacts to mitochondrial misfolding by reducing protein import and activating mitophagy. Nat. Commun. 13, 5164. (10.1038/s41467-022-32564-x) PMC9440083 36056001

[152] Li J , Chauve L, Phelps G, Brielmann RM, Morimoto RI. 2016 E2F coregulates an essential HSF developmental program that is distinct from the heat-shock response. Genes Dev. 30, 2062–2075. (10.1101/gad.283317.116) PMC5066613 27688402

[153] Tian Y , Garcia G, Bian Q, Steffen KK, Joe L, Wolff S, Meyer BJ, Dillin A. 2016 Mitochondrial stress induces chromatin reorganization to promote longevity and UPR^mt^. Cell 165, 1197–1208. (10.1016/j.cell.2016.04.011) PMC4889216 27133166

[154] Labbadia J , Brielmann RM, Neto MF, Lin YF, Haynes CM, Morimoto RI. 2017 Mitochondrial stress restores the heat shock response and prevents proteostasis collapse during aging. Cell Rep. 21, 1481–1494. (10.1016/j.celrep.2017.10.038) PMC5726777 29117555

[155] Berendzen KM , Durieux J, Shao LW, Tian Y, Kim HE, Wolff S, Liu Y, Dillin A. 2016 Neuroendocrine coordination of mitochondrial stress signaling and proteostasis. Cell 166, 1553–1563.e10. (10.1016/j.cell.2016.08.042) PMC5922979 27610575

[156] Shao LW , Niu R, Liu Y. 2016 Neuropeptide signals cell non-autonomous mitochondrial unfolded protein response. Cell Res. 26, 1182–1196. (10.1038/cr.2016.118) PMC5099867 27767096

[157] Zhang Q , Wu X, Chen P, Liu L, Xin N, Tian Y, Dillin A. 2018 The mitochondrial unfolded protein response is mediated cell-non-autonomously by retromer-dependent Wnt signaling. Cell 174, 870–883.e17. (10.1016/j.cell.2018.06.029) PMC6086732 30057120

[158] Kim KH *et al.* 2013 Autophagy deficiency leads to protection from obesity and insulin resistance by inducing Fgf21 as a mitokine. Nat. Med. 19, 83–92. (10.1038/nm.3014) 23202295

